# Beyond the regulatory radar: knowledge and practices of rural medical practitioners in Bangladesh

**DOI:** 10.1186/s12913-023-10317-w

**Published:** 2023-11-30

**Authors:** Hasnat Sujon, Mohammad Habibur Rahman Sarker, Aftab Uddin, Shakila Banu, Mohammod Rafiqul Islam, Md. Ruhul Amin, Md. Shabab Hossain, Md. Fazle Alahi, Mohammad Asaduzzaman, Syed Jafar Raza Rizvi, Mohammad Zahirul Islam, Md. Nazim Uzzaman

**Affiliations:** 1 International Centre for Diarrhoeal Disease Research, Bangladesh (icddr,b), Dhaka, Bangladesh; 2 Directorate General of Health Services, Ministry of Health and Family Welfare, Dhaka, Bangladesh; 3 Public Health Foundation of Bangladesh, Dhaka, Bangladesh; 4 faith Bangladesh, Dhaka, Bangladesh; 5 Translational Biology, Medicine, and Health Graduate Programme, Virginia Polytechnic Institute and State University, Blacksburg, VA USA; 6 University of Saskatchewan, Saskatoon, Canada; 7 The University of Queensland, Brisbane, Australia; 8 Usher Institute, The University of Edinburgh, Edinburgh, UK

**Keywords:** Bangladesh, Common cold, Community health workers, Diarrhoea, Pneumonia, Primary health care, Traditional medical practitioners

## Abstract

**Background:**

Informal and unregulated rural medical practitioners (RMPs) provide healthcare services to about two-thirds of people in Bangladesh, although their service is assumed to be substandard by qualified providers. As the RMPs are embedded in the local community and provide low-cost services, their practice pattern demands investigation to identify the shortfalls and design effective strategies to ameliorate the service.

**Methods:**

We conducted a cross-sectional study in 2015–16 using a convenient sample from all 64 districts of Bangladesh. Personnel practising modern medicine, without any recognized training, or with recognized training but practising outside their defined roles, and without any regulatory oversight were invited to take part in the study. Appropriateness of the diagnosis and the rationality of antibiotic and other drug use were measured as per the Integrated Management of Childhood Illness guideline.

**Results:**

We invited 1004 RMPs, of whom 877 consented. Among them, 656 (74.8%) RMPs owned a drugstore, 706 (78.2%) had formal education below higher secondary level, and 844 (96.2%) had informal training outside regulatory oversight during or after induction into the profession. The most common diseases encountered by them were common cold, pneumonia, and diarrhoea. 583 (66.5%) RMPs did not dispense any antibiotic for common cold symptoms. 59 (6.7%) and 64 (7.3%) of them could identify all main symptoms of pneumonia and diarrhoea, respectively. In pneumonia, 28 (3.2%) RMPs dispensed amoxicillin as first-line treatment, 819 (93.4%) dispensed different antibiotics including ceftriaxone, 721 (82.2%) dispensed salbutamol, and 278 (31.7%) dispensed steroid. In diarrhoea, 824 (94.0%) RMPs dispensed antibiotic, 937 (95.4%) dispensed ORS, 709 (80.8%) dispensed antiprotozoal, and 15 (1.7%) refrained from dispensing antibiotic and antiprotozoal together.

**Conclusions:**

Inappropriate diagnoses, irrational use of antibiotics and other drugs, and polypharmacy were observed in the practising pattern of RMPs. The government and other stakeholders should acknowledge them as crucial partners in the healthcare sector and consider ways to incorporate them into curative and preventive care.

**Supplementary Information:**

The online version contains supplementary material available at 10.1186/s12913-023-10317-w.

## Introduction

The better-than-expected health performance of Bangladesh in the last decades is dubbed as the “Bangladesh paradox”– indicating its success in dramatic reduction of mortality, despite the widespread presence of poverty, inequity, lack of utilization of basic health services, and uneven morbidities [1]. This exceptional achievement is due to a multitude of effects, one of them is the pluralistic health system– the effort of multiplicity of stakeholders through a combination of a centrally-planned government-controlled public health sector, and a *laisses-faire* system of informal health providers [2]. Informal health providers are a heterogenous group of unregistered healthcare providers usually without any formally recognized training but may receive informal training, who accept payment directly from the patients and work outside government regulation [3], however, there is no single definition that fits all types of informal providers. In Bangladesh, they practice both traditional and modern medicine and are composed of several cadres of providers such as traditional healers, birth attendants, village doctors (*polli chikitshok)*, drug store salespersons etc. [2]. There is considerable overlap between the roles of these cadres. For example, a village doctor can also work as a drug store salesperson and vice versa. Personnel who were trained as community health workers (CHW) by the government or a non-government organization or trained as a paraprofessional such as medical assistants also can own a drug store, and work beyond their regulatory limit as an informal health provider. The cadres of informal health providers who practice modern medicine viz. village doctors, drug store salespersons, CHWs, and medical assistants are loosely termed rural medical practitioners (RMPs) or village doctors when they provide consultation to patients, although the concept of village doctor started in Bangladesh in the 1980s imitating the ‘barefoot doctor’ of Mao’s China [4]. Since the beginning of this initiative in Bangladesh, the Bangladesh Rural Medical Practitioner Training (BRMP) has become a popular training and qualification for RMPs. However, now an extensive amount of training is available for RMPs, most of which are not regulated by the government.

The idea behind the current health infrastructure of Bangladesh was to develop a pro-poor community-based health system [5]; however, evidence suggests that the poor are functionally excluded unless services are “geographically accessible, of decent quality, fairly financed and responsive” [6]. The state-sponsored health sector is festered with a prevailing inequity of deployment of the workforce in terms of geographical location, gender sensitiveness and skill mix [2, 4]. In addition, there is a critical health provider shortage in the entire health workforce with < 10 health workers (dentists, medical doctors, midwifery personnel, nursing personnel, pharmacists) per 10,000 population [2, 7]. This shortage of qualified providers and their inaccessibility propels people, particularly the poor and the disadvantaged, towards seeking care from the RMPs. Other barriers such as financial hardship, lack of access to information on available services, cultural factors prohibiting females from seeking medical care from a male provider etc. also facilitate people to seek medical care from the informal sector [8–10]. 60–77% of all healthcare services accessed in Bangladesh are provided by the RMPs [3], although some of these services can be considered illegal under the current regulations in Bangladesh. For example, dispensing antibiotics without a prescription from a certified physician is prohibited under the Drugs and Cosmetics Act 2023 [11], and only a limited prescribing is permitted from a pre-specified list by the community health staff working directly for the government which is specified in their job descriptions [12–14]. Despite these regulations, dispensing drugs for all diseases including communicable and non-communicable diseases outside the regulatory limit is ubiquitous in Bangladesh.

RMPs are trained in communicable diseases that are prevalent throughout the country, such as diarrhoea, common cold, acute respiratory infection (ARI) /pneumonia etc. Bangladesh, with four other nations, accounts for half of all paediatric pneumonia cases worldwide with about two million pneumonia cases diagnosed each year [15]. In recent years, Bangladesh saw a decline in under 5 diarrhoea and ARI cases, with an overall prevalence of 4.9% and 3.0%, respectively [16].

Given the RMPs are rooted in the local community, provide affable low-cost health care, and the most popular health providers in Bangladesh [2, 8, 10], it is imperative that their practice pattern, particularly for communicable diseases should be analysed to identify the shortfalls and design effective intervention to overcome the challenges. Healthcare provided by the RMPs is generally perceived as substandard by formal healthcare providers such as registered physicians [3] and there is an apprehension toward this informal sector among qualified providers [9]. Very few studies have been conducted on RMPs in Bangladesh exploring their background dynamics and dispensing patterns, however, none of them utilized nationwide data [8–10, 17–19]. Only one study in 2007 presented nationwide data to evaluate the distribution of providers and their practising pattern but reported only descriptive analysis [4]. A telephone survey, conducted in 2019, explored the characteristics of RMPs [20]. In this study, we aimed to evaluate the practice pattern of RMPs in handling three common diseases encountered by them viz. common cold, pneumonia and diarrhoea, and their associated factors using data collected from the entire country.

## Methodology

### Study settings and population

The International Centre for Diarrhoeal Disease Research, Bangladesh (icddr,b) is an international health research organisation in Dhaka, Bangladesh. The Technical Training Unit of icddr,b arranged a five-day capacity-building programme for the RMPs in 2014–16 to educate them on diagnosing and managing the commonest diseases encountered by the RMPs. From all districts of Bangladesh, the funding agency of the programme conveniently selected the trainee RMPs having three criteria: (1) practice modern medicine, (2) without any recognized training, or with recognized training, but practising outside their defined roles (3) without any regulatory oversight. However, personnel formally employed by the government or any non-government organization despite meeting all three criteria were not included in the training. From March 2015 to October 2016, we conducted this cross-sectional study using a convenient sample by inviting all trainees (1004) during this period to participate in the study before attending the training programme, among which 877 RMPs completed the survey questionnaire.

### Method of data collection

We used a semi-structured two-part questionnaire (Additional file 1) to collect the data and the interview was conducted by trained registered physicians. The first part of the questionnaire was about the characteristics of the RMPs (i.e., practice site, educational qualification, years of experience, training received, available facilities at the practice site) and the names of the common diseases treated by them. The second part of the questionnaire collected data on identifying signs and treatment of the three commonest diseases encountered (i.e., common cold, pneumonia, and diarrhoea) by the RMPs. Prior to data collection, we pre-tested the questionnaire on a convenient sample of 25 RMPs. The questionnaire was amended as per the responses received during the pre-testing.

### Statistical analysis

We performed a descriptive analysis of the characteristics and practice patterns of the respondents. We also analysed the association of the appropriate diagnosis and rational treatment of the common cold, pneumonia, and diarrhoea with their characteristics and performed the Pearson chi-square test or Fisher’s exact test (when more than 20% of cells have frequencies below 5) to measure the association. A *p*-value of < 0.05 was considered statistically significant. The analysis, including the significance test, was performed using STATA version 17 (StataCorp).

We used Integrated Management of Childhood Illness (IMCI) criteria [21] to measure the appropriateness of diagnosis of pneumonia and diarrhoea, and the rationality of dispensing antibiotics and other drugs for common cold, pneumonia, and diarrhoea.

## Results

**Fig. 1:**
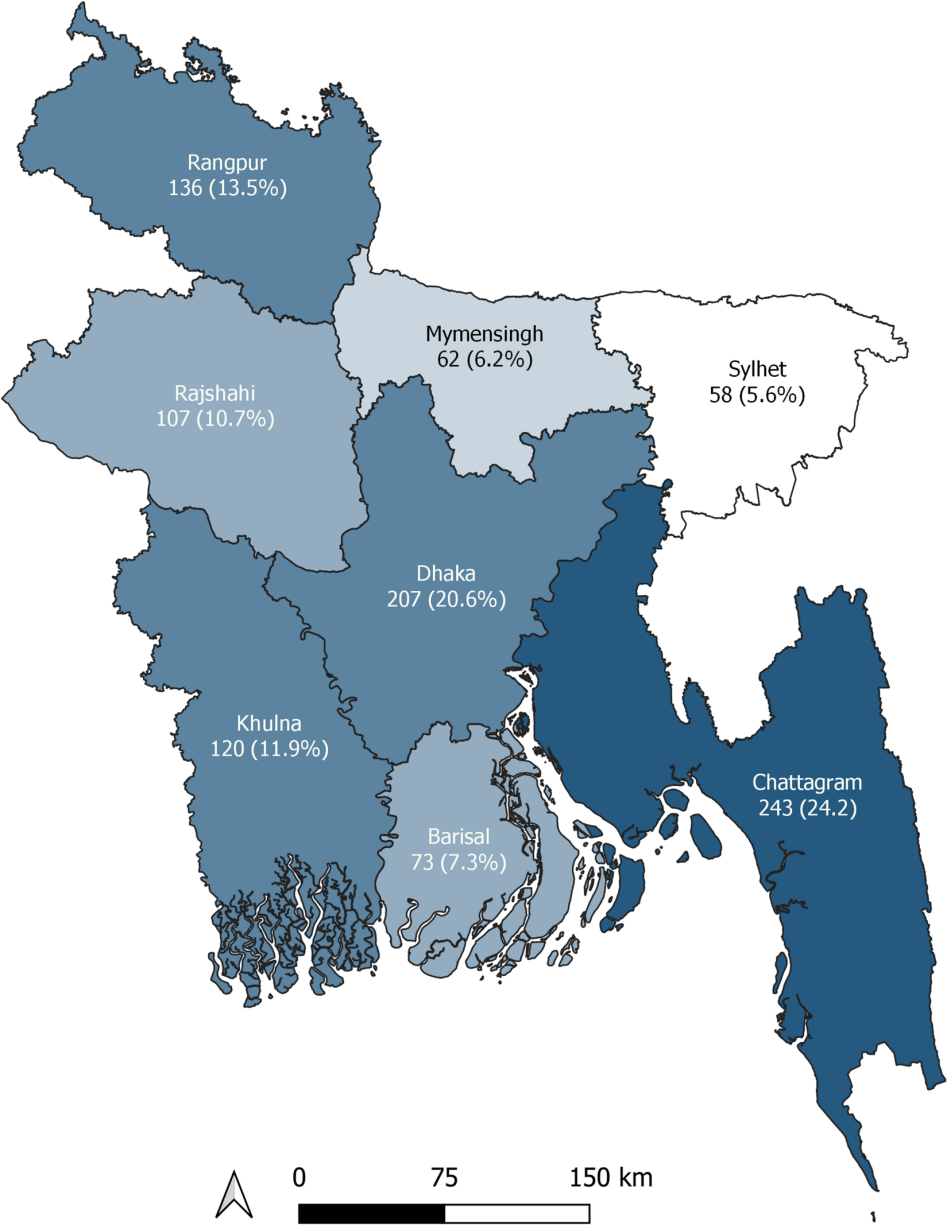
Distribution of the respondents (*N* = 877)

All respondents were male. Most of the participants were from Chattagram division, comprising one-quarter (25.8%) of the total participants, followed by Dhaka division (20%). Sylhet division accounted for the lowest number of respondents (6%) (Fig. 1).

Three-quarters (74.8%) of the respondents owned a drugstore and worked as a drugstore salesperson while working as an RMP. Only 19.5% of them had formal schooling above higher secondary level, and almost half of them attended only up to secondary school. Most (67.4%) of the respondents had ≥ 10 years’ experience as an RMP. Regarding training, 3.8% attended any government-supervised training course. Among the training attended by the RMPs, Local Medical Assistant & Family Planning (LMAFP) training and Bangladesh Rural Medical Practitioner (BRMP) training were the most popular. A list of available training is reported in Additional file 2. The three most common diseases encountered by the RMPs were common cold, pneumonia, and diarrhoea (Table 1).

**Table 1:** Characteristics of the rural medical practitioners and the commonest diseases treated by them

Characteristics (*N* = 877)	n (%)
**Gender**	
Male	877 (100.0)
**Practice site (multiple response)**	
Own chamber^a^	156 (17.8)
Own drugstore	656 (74.8)
Other's drugstore	66 (7.5)
Own house	10 (1.1)
Others	3 (0.3)
**Educational qualification**	
Masters (science)	9 (1.0)
Masters (other than science)	22 (2.5)
Bachelor (science)	48 (5.5)
Bachelor (other than science)	92 (10.5)
Higher secondary school or equivalent	274 (31.2)
Secondary school or equivalent	412 (47.0)
Below secondary school	20 (2.3)
**Experience (years)**	
1–9	286 (32.6)
10–19	401 (45.7)
≥ 20	190 (21.7)
**Training (multiple response)**	
Training course	
LMAFP	335 (38.2)
BRMP	375 (42.8)
Others^b^	196 (22.4)
Government oversight	
At least one training under government oversight	33 (3.8)
Training without government oversight	844 (96.2)
**Commonest diseases treated**	
Common cold	709 (80.8)
Pneumonia	51 (9.2)
Diarrhoea	50 (5.7)
Hypertension	19 (2.1)
Diabetes	14 (1.6)
Asthma	4 (0.5)

*LMAFP* Local Medical Assistant & Family Planning training, *BRMP* Bangladesh Rural Medical Practitioner training

^a^Private consultation

^b^For a list of other training, see Additional file 1

For the management of the common cold, two-thirds (66.5%) of the respondents did not dispense any antibiotics. Among the respondents who used antibiotics, azithromycin and amoxicillin were the most popular antibiotics (Additional file 3). For the diagnosis of pneumonia, at least half of them knew that difficulty in breathing (76.3%), chest indrawing (57.6%), and fever (54.5%) were the signs of pneumonia. Almost three-quarters (73.9%) dispensed multiple antibiotics (both oral and injectable) and salbutamol (82.2%) for pneumonia, and one-third (31.7%) dispensed steroids. More than two-thirds knew sunken eyes (76.5%), delayed skin pinch (69.4%) and increased thirst (66.8%) as the signs of dehydration. Almost all of them dispensed antibiotics (94.0%) and oral rehydration saline (ORS) (95.4%) for the treatment of diarrhoea. > 80% also dispensed intravenous cholera saline and antiprotozoal such as metronidazole (Table 2).

**Table 2:** Practice pattern of the rural medical practitioners in case of commonest disease treated

Characteristics (*N* = 877)	n (%)
**Common cold**	
Treatment of common cold	
Do not dispense antibiotic (rational use)	583 (66.5)
Dispense multiple antibiotics	120 (13.7)
Dispense single antibiotic	174 (19.8)
**Pneumonia**	
Diagnosis/ signs of pneumonia (multiple response)	
Cough	338 (38.5)
Difficulty in breathing	669 (76.3)
Fast breathing	349 (39.8)
Chest indrawing	505 (57.6)
Fever	478 (54.5)
Others	353 (40.3)
Treatment of pneumonia (multiple response)	
Antibiotic	
Do not dispense antibiotic	30 (3.4)
Dispense amoxicillin as first line of treatment (rational use)	28 (3.2)
Dispense ceftriaxone as first line of treatment	171 (19.5)
Dispense various antibiotics (both oral and injectable)	648 (73.9)
Salbutamol	721 (82.2)
Steroid	278 (31.7)
Antihistamine	402 (45.8)
Others	440 (50.2)
Referral in severe cases	827 (94.3)
**Diarrhoea**	
Diagnosis/ signs of dehydration (multiple response)	
Lethargic appearance	257 (29.3)
Sunken eyes	671 (76.5)
Increased thirst	586 (66.8)
Delayed skin pinch	609 (69.4)
Others	183 (20.9)
Treatment of diarrhoea (multiple response)	
Oral rehydration saline	837 (95.4)
Cholera saline	741 (84.5)
Antibiotic	824 (94.0)
Antiprotozoal	709 (80.8)
Antiemetic	600 (68.4)
Others	253 (28.8)
Referral in severe case	779 (88.8)

Only ~ 7% of the respondents could identify three major signs of pneumonia (cough, fast breathing, and chest indrawing), and four major signs of dehydration (lethargic appearance, sunken eyes, delayed skin pinch, and increased thirst) together. Although most of them (66.5%) used antibiotics rationally in case of common cold (i.e., did not use any antibiotic), only 3.2% resorted to dispensing amoxicillin as the first line treatment in pneumonia, and only 6.0% refrained from dispensing any antibiotic in diarrhoea. In the case of salbutamol and steroid in pneumonia, and ORS and antiprotozoal in diarrhoea, > 80% used a rational approach. We found that appropriate diagnosis of pneumonia and diarrhoea were significantly associated with years of experience (*p* = 0.02 and < 0.01, respectively), rational use of antibiotics in common cold was significantly associated with geographical distribution (*p* =  < 0.01), and rational use of salbutamol and steroid in pneumonia were significantly associated with both geographical distribution (*p* = 0.03 and < 0.01, respectively) and experience (*p* = 0.04 and 0.04, respectively). However, we did not find any association of the rational use of antibiotics in pneumonia and diarrhoea, rational use of ORS and antiprotozoal in diarrhoea with any of the characteristics of the RMPs (Table 3).

**Table 3:** Characteristics of rural medical practitioners and their association with appropriate diagnosis and rational treatment of different diseases

Characteristics (*N* = 877)	n (%)	Appropriate diagnosis of				Rational use of antibiotic in						Rational use of other drugs							
		**pneumonia**^**a**^		**dehydration**^**b**^		**common cold**^**c**^		**pneumonia**^**d**^		**diarrhoea**^**e**^		**salbutamol in pneumonia**^**f**^		**steroid in pneumonia**^**g**^		**ORS in diarrhoea**^**h**^		**antiprotozoal in diarrhoea**^**i**^	
		**n (%)**	***p* value**	**n (%)**	***p* value**	**n (%)**	***p* value**	**n (%)**	***p* value**	**n (%)**	***p* value**	**n (%)**	***p* value**	**n (%)**	***p* value**	**n (%)**	***p* value**	**n**	***p* value**
**Division**																			
Barisal	62 (7.0)	1 (0.1)	0.23	4 (0.5)	0.79	45 (5.1)	** < 0.01**	3 (0.3)	0.45	3 (0.3)	0.11	44 (5.0)	**0.03**	11 (1.3)	** < 0.01**	60 (6.8)	0.21	0 (0.0)	0.15
Chattagram	226 (25.8)	18 (2.1)		13 (1.5)		154 (17.6)		5 (0.6)		8 (0.9)		196 (22.3)		91 (10.4)		216 (24.6)		4 (0.5)	
Dhaka	179 (20.4)	19 (2.2)		11 (1.3)		97 (11.1)		6 (0.7)		10 (1.1)		142 (16.2)		48 (5.5)		173 (19.7)		3 (0.3)	
Khulna	102 (11.6)	6 (0.7)		9 (1.0)		64 (7.3)		2 (0.2)		9 (1.0)		79 (9.0)		37 (4.2)		93 (10.6)		0 (0.0)	
Mymensingh	56 (6.4)	3 (0.3)		4 (0.5)		38 (4.3)		4 (0.5)		1 (0.1)		43 (4.9)		20 (2.3)		52 (5.9)		0 (0.0)	
Rajshahi	92 (10.5)	4 (0.5)		10 (1.1)		58 (6.6)		3 (0.3)		6 (0.7)		79 (9.0)		33 (3.8)		90 (10.3)		1 (0.1)	
Rangpur	112 (12.8)	7 (0.8)		10 (1.1)		90 (10.3)		5 (0.6)		13 (1.5)		98 (11.2)		25 (2.9)		109 (12.4)		6 (0.7)	
Sylhet	48 (5.5)	1 (0.1)		3 (0.3)		36 (4.1)		0 (0.0)		3 (0.3)		40 (4.6)		13 (1.5)		44 (5)		1 (0.1)	
**Educational qualification**																			
Secondary school or below	432 (49.3)	28 (3.2)	0.30	41 (4.7)	0.91	293 (33.4)	0.48	13 (1.5)	0.87	27 (3.1)	0.45	367 (41.8)	0.11	136 (15.5)	0.5	414 (47.2)	0.68	7 (0.8)	0.33
Higher secondary school or equivalent	274 (31.2)	23 (2.6)		13 (1.5)		174 (19.8)		10 (1.1)		13 (1.5)		219 (25.0)		93 (10.6)		259 (29.5)		3 (0.3)	
Bachelor or Masters	171 (19.5)	8 (0.9)		10 (1.1)		115 (13.1)		5 (0.6)		13 (1.5)		135 (15.4)		49 (5.6)		164 (18.7)		5 (0.6)	
**Training**																			
Training course (multiple response)																			
LMAFP (yes)	335 (38.2)	25 (2.9)	0.49	24 (2.7)	0.38	210 (23.9)	0.07	11 (1.3)	0.90	17 (1.9)	0.34	270 (30.8)	0.33	114 (13.0)	0.24	317 (36.1)	0.37	6 (0.7)	0.80
LMAFP (no)	542 (61.8)	852 (57.1)		853 (97.3)		667 (76.1)		866 (98.7)		860 (98.1)		607 (69.2)		763 (87.0)		560 (63.9)		871 (99.3)	
BRMP (yes)	375 (42.8)	24 (2.7)	0.74	24 (2.7)	0.38	259 (29.5)	0.14	11 (1.3)	0.71	27 (3.1)	0.21	315 (35.9)	0.23	112 (12.8)	0.31	359 (40.9)	0.72	9 (1.0)	0.14
BRMP (no)	502 (57.2)	853 (97.3)		853 (97.3)		618 (70.5)		866 (98.7)		850 (96.9)		562 (64.1)		765 (87.2)		518 (59.1)		868 (99.0)	
Others (yes)	196 (22.3)	12 (1.4)	0.70	17 (1.9)	0.40	131 (14.9)	0.87	6 (0.7)	0.91	12 (1.4)	0.96	160 (18.2)	0.81	64 (7.3)	0.75	186 (21.2)	0.68	3 (0.3)	0.88
Others (no)	681 (77.7)	865 (98.6)		860 (98.1)		746 (85.1)		871 (99.3)		865 (98.6)		717 (81.8)		813 (92.7)		691 (78.8)		874 (99.7)	
Government oversight																			
At least one training under government oversight	33 (3.8)	2 (0.2)	0.88	1 (0.10)	0.38	24 (2.7)	0.43	1 (0.1)	0.96	2 (0.2)	1.00	31 (3.5)	0.07	9 (1.0)	0.58	32 (3.6)	0.67	0 (0.0)	0.57
Training without government oversight	844 (96.2)	57 (6.5)		63 (7.2)		558 (63.6)		27 (3.1)		51 (5.8)		690 (78.7)		269 (30.7)		805 (91.8)		15 (1.7)	
**Experience (years)**																			
1–9	286 (32.6)	14 (1.6)	**0.02**	9 (1.0)	** < 0.01**	184 (21)	0.08	8 (0.9)	0.39	15 (1.7)	0.76	222 (25.3)	**0.04**	222 (25.3)	**0.04**	273 (31.1)	0.53	6 (0.7)	0.39
10–19	401 (45.7)	37 (4.2)		34 (3.9)		259 (29.5)		11 (1.3)		25 (2.8)		341 (38.9)		341 (38.9)		380 (43.3)		8 (0.9)	
≥ 20	190 (21.7)	8 (0.9)		21 (2.4)		139 (15.8)		9 (1.0)		13 (1.5)		158 (18.0)		158 (18.0)		184 (21.0)		1 (0.1)	
**Total**	877 (100.0)	59 (6.7)		64 (7.3)		583 (66.5)		28 (3.2)		53 (6.0)		721 (82.2)		278 (31.7)		837 (95.4)		15 (1.7)	

*ORS* Oral rehydration saline, *LMAFP* Local Medical Assistant & Family Planning training, *BRMP* Bangladesh Rural Medical Practitioner Training. For calculating the percentage in each category, total sample size (877) was used as the denominator. Pearson’s chi-square test or Fisher’s exact test (when more than 20% of cells have frequencies below 5) was performed to measure association

^a^Appropriate diagnosis of pneumonia means at least knowing cough, fast breathing, and chest indrawing together

^b^Appropriate diagnosis of dehydration means at least knowing lethargic appearance, sunken eyes, delayed skin pinch and increased thirst together

^c^Rational use of antibiotics in common cold means do not dispense antibiotic

^d^Rational use of antibiotics in pneumonia means dispensing amoxicillin as the first line therapy

^e^Rational use of antibiotics in diarrhoea means do not dispense antibiotic

^f^Rational use of salbutamol in pneumonia means dispensing salbutamol

^g^Rational use of steroids in pneumonia means do not dispense steroid

^h^Rational use of ORS in diarrhoea means dispensing ORS

^i^Rational use of antiprotozoal in diarrhoea means do not dispense antibiotic and antiprotozoal together

## Discussion

We gathered data from all districts in Bangladesh to understand the knowledge and practice patterns of RMPs. The majority of RMPs in Bangladesh own and operate drugstores. Their formal education is usually below higher secondary school, followed by training without government oversight. In terms of case management, despite prevailing regulations, RMPs in Bangladesh commonly dispense various drugs, including antibiotics, without a prescription from a registered physician. Our findings indicate that only a small number of RMPs demonstrate the ability to accurately diagnose and prescribe antibiotics and other medications for conditions like pneumonia and diarrhoea.

Previous studies have categorized RMPs into different groups, including village doctors (who received informal training during induction to the profession), drugstore salespersons (who were inducted into the profession through selling medicines or apprenticeships), paraprofessionals such as medical assistants and CHWs [4, 19]. However, it's challenging to classify them strictly, as one person may fit multiple definitions. Some studies even included individuals working within regulatory limits (e.g., formal employees of government or non-government organizations) in the RMP category if they offer consultations beyond their defined job description, like CHWs and medical assistants [4]. In our study, we only considered CHWs and medical assistants who don't work formally for government or non-government organizations. In addition, previous studies have used various terms for RMPs, like 'informal health providers', 'village doctors', or 'unqualified health providers' [3, 4, 8–10, 19]. However, there is no unanimous definition of these terms, and each has its own limitations. In our study, despite the term ‘RMPs’ might convey the message that all participants were rural, some of our participants were, in fact, not living in rural areas. After entering the profession, RMPs typically undergo one or more informal training. Bangladesh offers a multitude of informal, unregulated training options for RMPs, with LMAFP and BRMP being the most popular, possibly due to their shorter duration and greater availability compared to government-regulated courses. The content, duration, and assessment methods of these courses vary among different provider organizations.

Respiratory diseases and diarrhoea are two major causes of childhood mortality in Bangladesh [22], and the RMPs encountered patients with common cold, pneumonia, and diarrhoea most often, which was reported in other studies too [4, 19]. The IMCI guidelines identified cough, fast breathing, and chest indrawing as the main signs of pneumonia, and lethargic appearance, sunken eyes, delayed skin pinch, and increased thirst as the main signs of dehydration [21]. Only ~ 7% of the respondents could identify these symptoms together, and this was associated with years of experience. More experienced RMPs could better identify all the symptoms together, which may indicate that the quality of the informal training RMPs received was sub-optimal, therefore recently trained RMPs missed the signs, and experience helped the RMPs to develop a ‘clinical eye’.

The IMCI guideline recommends not using any antibiotic for common cold and using amoxicillin as the first-line treatment for pneumonia [21]. In the case of diarrhoea, antibiotic is recommended only in selected cases such as dysentery [21]. We found that although most of the RMPs conformed to the recommendation in the case of common cold, almost all of them dispensed various antibiotics in case of pneumonia and diarrhoea. This was reported in other studies too [3, 4, 8, 9, 17–19]. We did not find any association between the rational use of antibiotics in pneumonia and diarrhoea, and the independent variables, which may be because almost all RMPs were dispensing antibiotics indiscriminately. This irrational use of antibiotics may fuel the growing prevalence of antimicrobial resistance in Bangladesh [23]. Regarding salbutamol use in pneumonia and ORS use in diarrhoea, almost all respondents conformed to the recommended practice. However, steroids in pneumonia should only be prescribed by a registered physician, which is disregarded by most of the RMPs. Another concerning aspect is polypharmacy, which has also been noted in other studies [4, 8, 9, 17–19]. We observed this in the case of diarrhoea management, where RMPs often dispensed antiprotozoal treatment alongside antibiotics in nearly all cases. The reason behind this lack of rational use of drugs might be a lack of knowledge, absence of any proper guidelines, unavailability of information in Bangla, and the incentives from pharmaceutical companies [24, 25].

RMPs face criticism from formally trained healthcare providers for potential misdiagnoses and unsafe practices [3, 9, 10]. While our study highlights instances of inappropriate diagnoses, polypharmacy, and medication dispensing beyond regulatory boundaries, it's important to recognize that locally established RMPs serve as the initial healthcare contact in all corners of the country, especially for rural communities. Their contribution is also appreciable for some positive changes such as very low levels of post-partum sepsis and virtual disappearance of rheumatic heart disease in Bangladesh [2]. Additionally, the severe shortage of qualified providers in rural areas, coupled with high absenteeism rates and a preference for urban practice [2, 4, 8, 10] leaves the rural poor with little to no option, but to seek care from the RMPs. Therefore, it is essential for the formal sector to devise a strategy to train and utilize them, instead of leaving them alone.

The proliferation of informal healthcare providers has outstripped the government's regulatory capacity due to rapid population and market growth [26]. Unfortunately, at the policy level, there is a near total blindness to this growing sector of informal cadres, which is evident from the National Health Policy 2011 of the government of Bangladesh [27]. While the policy emphasizes universal primary healthcare, it overlooks strategies to recognize and address the significant role played by RMPs in healthcare delivery [2, 4]. The National Health Policy 2011 [27], Bangladesh Health Workforce Strategy 2015 [28], and Bangladesh National Strategy for Community Health Workers 2019–2030 [29] suggest an intention to augment the workforce with more CHWs, both from government and non-government organizations, to meet community-level healthcare needs. Government-regulated CHWs include Health Assistants, Family Welfare Assistants, and Community Health Care Providers, offering curative and preventive services at the community level [29]. The utilization of local CHWs has been instrumental in Bangladesh's notable achievements in health indicators like reduced maternal and infant mortality rates [30]. However, CHWs currently constitute a small fraction of the overall health workforce [2]. The overinflated expectation of the government that a centrally controlled health infrastructure can provide healthcare services to a population as big as Bangladesh is presumptuous at best, overlooking the reality that the vast majority of the population seeks healthcare from the RMPs [2], and the rapid growth of these informal healthcare providers outpacing the regulatory capacity of the government [26]. We must acknowledge the fact that this health-seeking behaviour does not occur in a vacuum, but as a complex function of various factors such as the ability of the consumers, availability and accessibility of healthcare providers, as well as cultural factors [8–10].

Given their widespread presence and the stake they hold in the healthcare market, it is only logical to incorporate the RMPs within a regulatory framework and devise interventions for healthy medical practice. This would be particularly beneficial given the critical shortage of healthcare professionals in Bangladesh [1, 7]. Task-shifting to trained RMPs can also alleviate the burden on qualified professionals. Regulations need to be devised not only to draw a line between wrong and right, but also to foster the potential of the RMPs, and to maintain the integrity and trust between formal and informal communities [2]. Interventions like training and supportive supervision have proven effective in improving correct case management, though they may not entirely eliminate polypharmacy [31]. There are also successful examples of projects aimed at improving the preventive and curative services provided by the RMPs in Bangladesh and India [32–35]. The "Model Pharmacy" programme, launched in 2016 by the Directorate General of Drug Administration, is a commendable effort to regulate drugstore salespersons and curb improper drug dispensing [36]. Promising endeavours using mHealth technology to connect RMPs with qualified professionals for improved patient care have also been documented [37].

Limitations of this study include its cross-sectional design with convenient participant sampling during a training course, potentially resulting in underrepresentation of certain districts. The assessment focused on reported practices, which may differ from actual practices due to participants' inclination to answer the questionnaire accurately. In addition, it would have been valuable to investigate the rationality of the treatment of common diseases by urban/rural locations. Analysing the root cause of the inappropriate diagnosis and irrational use of drugs might help devise interventions to improve the practising pattern of the RMPs, which was beyond the scope of this study. We also did not analyse the curricula of different training courses, which may be an attempt to standardize the training, and in turn, brought them under regulation. A big nationwide data set was the main strength of the study.

## Conclusion

Although their practising pattern is riddled with inadequacy and inappropriateness, RMPs are responsible for the majority of the healthcare provided to the citizens of Bangladesh. Government and other relevant stakeholders should devise interventions to ameliorate the service provided by them. The first step of such an active strategy would be to recognize them as an important stakeholder in the healthcare community. Given their widespread presence within the local community all over the country, incorporating them in preventing and curative services through regulation, training, and monitoring will bring long-term positive health outcomes.

### Supplementary Information

**Additional file 1.**

**Additional file 2.**

**Additional file 3.**

## Data Availability

The detailed dataset is available with the corresponding author of this article. A copy of the original data is also stored in the data archive of icddr,b. The datasets used and analysed during the current study are available from the corresponding author upon reasonable request, subject to the approval of the Research Administration of icddr,b.

## Abbreviations

BRMP: Bangladesh Rural Medical Practitioner training
CHWs: Community Health Workers
IMCI: Integrated Management of Childhood Illness
LMAFP: Local Medical Assistant & Family Planning training
ORS: Oral rehydration saline
RMPs: Rural medical practitioners
