# Supplementary material for: Beyond the regulatory radar: knowledge and practices of rural medical practitioners in Bangladesh
Source: BMC Health Serv Res. 2023 Nov 30;23:1322. doi: 10.1186/s12913-023-10317-w (PMC10688090; doi:10.1186/s12913-023-10317-w)
Supplement: Supplementary file 1 — Additional file 1. [file 12913_2023_10317_MOESM1_ESM.pdf]

Additional File 1. Questionnaire used to collect data

Part 1. Information on Socio-Demographics:

1. Respondent's ID:
2. Age (years): ____________
3. Sex: ☐Male ☐Female
4. Address: Village:

   Union:

   Upazilla:

   District:
5. Education

   ☐Below SSC/secondary school (please specify years of schooling)_______

   ☐SSC/ Secondary school or equivalent

   ☐HSC/ Higher secondary school or equivalent

   ☐Bachelor (please mention the subject of study)__________

   ☐Masters (please mention the subject of study)__________
6. Professional qualification (multiple response allowed)

   ☐LMAF ☐BRMP ☐DMF/MATS ☐Paramedical

   ☐Palli Chikitshok (PC) ☐Others (Please specify)___________________
7. Duration of practice (in year): _________
8. Rank the following diseases on a scale of 1-10 depending on your encounter (the disease you encounter most should be ranked 1, and the least should be ranked 10)

   Diarrhoea _______

   Diabetes _______

   Common cold _______

Hypertension ________

Pneumonia ________

Asthma ________

Typhoid ________

Trauma ________

UTI ________

Others ________ please specify___________

9. What are the tools you have now in your chamber for consulting with a patient? (multiple response)

☐Stethoscope ☐BP machine ☐Weight machine ☐Torch

☐Thermometer ☐Nebulizer ☐Glucometer

☐Surgical instruments for stitching ☐Arrangement for handwashing

Part 2. Practice pattern of rural medical practitioners

1. Where do you usually consult patients?

   ☐Own chamber ☐Own drugstore ☐Other's drugstore ☐Own house

   ☐Others (please specify)_____________

2. How many patients do you consult on average in a day?_______

3. How do you treat common cold?

   ☐I do not prescribe antibiotics for common cold

   ☐I prescribe a single antibiotic (please specify)___________

   ☐I prescribe multiple antibiotics (please specify)____________

4. Which of the following are the signs of pneumonia (multiple response possible)

   ☐Cough ☐Difficulty breathing ☐Fast breathing ☐Fever

   ☐Chest indrawing ☐Other (please specify)__________________

5. How do you treat pneumonia? (multiple response)

   ☐I do not prescribe antibiotics for pneumonia

   ☐I prescribe antibiotics for pneumonia

   - ☐I prescribe amoxicillin as the first line of treatment
   - ☐I prescribe ceftriaxone as the first line of treatment
   - ☐I prescribe various antibiotics both oral and injectables (please specify)_________________________________

   ☐I prescribe salbutamol for pneumonia

   ☐I prescribe steroids for pneumonia (please specify)______________

   ☐I prescribe antihistamine for pneumonia

   ☐I prescribe other drugs for pneumonia (please specify)_____________

   ☐I refer severe cases to hospital or qualified providers

6. Which of the following are signs of dehydration?

☐Lethargic appearance ☐Sunken eyes ☐Thirsty

☐Delayed skin pinch ☐Others (please specify)______________

7. How do you treat diarrhoea? (multiple response)

☐I prescribe ORS for diarrhoea

☐I prescribe cholera saline for diarrhoea

☐I prescribe antibiotics for diarrhoea (please specify)______________

☐I prescribe antiprotozoal for diarrhoea (please specify)____________

☐I prescribe both antibiotic and antiprotozoal together for diarrhoea

☐I prescribe antiemetic for diarrhoea (please specify)________________

☐I prescribe other drugs for diarrhoea (please specify)_______________

☐I refer severe cases to hospital or qualified provider
