# Supplementary material for: Beyond the regulatory radar: knowledge and practices of rural medical practitioners in Bangladesh
Source: BMC Health Serv Res. 2023 Nov 30;23:1322. doi: 10.1186/s12913-023-10317-w (PMC10688090; doi:10.1186/s12913-023-10317-w)
Supplement: Supplementary file 2 — Additional file 2. [file 12913_2023_10317_MOESM2_ESM.pdf]

Additional file 2 Training courses taken by the rural medical practitioners.

Name of the training	Duration
**Government appoved training**[a]	
Diploma of Medical Faculty/ Medical Asstistant Training (DMF)	3 years
Community Health Wrokers (CHWs)[b]	Variable
Diploma in Dental Technology (DDT)	3 years
Diploma in Intensive Care Assistant (DICA)	3 years
Diploma in Medical Laboratory Technology (DMLT)	3 years
Diploma in Medical Technology (Physiotherapy)(DMT)	3 years
Diploma in Medical Technology (Radiography) (DMT)	3 years
Diploma in Medical Technology (Radiotherapy) (DMT)	3 years
Diploma in Operation Theatre Assistant (OTA)	3 years
Diploma Occupational Therapist (DOT)	3 years
Diploma in Homoeopathic Medicine & Surgery (DHMS)	4 years
Diploma in Unani Medicine & Surgery (DUMS)	4 years
**Training courses beyond government oversight**[c]	
Local Medical Assistant & Family Planning (LAMFP)	6, 8, or 12 months
Bangladesh Rural Medical Practitioner (BRMP)	3, 6 or 12 months
Certificate in Nursing	1 year
Certificate in Paramedics	1 year
Certificate in Pathology	1 or 2 years
Certificate in Physiotherapy	1 or 2 years
Cetrificate in Dentistry	1 year
Community Medical Assistant Certificate Course	3, 6 or 12 months
Diploma in Dentistry	3 or 4 year
Diploma in Dental Assistance	2 years
Diploma in Medical Assistance (DMA)	1, 2 or 3 years
Diploma in Medicine and Surgery (DMDS)	4 years
Diploma in Nursing	2, 3, or 4 years
Diploma in Paramedical course (DPM)	2 years
Diploma in Pathology	3 or 4 years
Diploma in Pharmacy	2 or 3 years
Diploma in Physiotherapy	3 or 4 years
Emergency Knowledge of Medical Science	9 months
Local Diploma in Medical Science	1 year
Pharmacy course	3 or 6 months, 1 or 2 years

[a]Personnel taking government-approved training is supposed to work within a regulatory limit. We considered personnel taking government-approved training as a rural medical practitioner, only when they worked beyond their regulatory limit and were not formally employed by the government or any non-government organization.
[b]Duration of the CHW training is variable depending on the cadres. Training is also provided by non-government organizations. Several cadres are included under the umbrella term of CHW, i.e., Health Assistants, Family Welfare Assistants, Community Health Care Providers, and non-government organizations' trained CHW.
[c]The list may not be exhaustive as these trainings are not under any monitoring.
