# Supplementary material for: Beyond the regulatory radar: knowledge and practices of rural medical practitioners in Bangladesh
Source: BMC Health Serv Res. 2023 Nov 30;23:1322. doi: 10.1186/s12913-023-10317-w (PMC10688090; doi:10.1186/s12913-023-10317-w)
Supplement: Supplementary file 3 — Additional file 3. [file 12913_2023_10317_MOESM3_ESM.pdf]

Additional file 3 Prescribed antibiotic in case of common cold by the rural medical practitioners.

**Antibiotic prescribed (N=294) (multiple response)**	**n (%)**
Azithromycin	153 (52.0)
Amoxicillin	128 (43.5)
Cefixim	75 (25.5)
Cefuroxim	44 (15.0)
Ciprofloxacin	35 (11.9)
Levofloxacin	26 (8.6)
Doxycyclin	4 (1.4)
Cephalexin	3 (1.0)
Penicillin	1 (0.3)
Cefpodoxime	1 (0.3)
Cephradine	1 (0.3)
Tetracycline	1 (0.3)
